# Medical Symbolism, in Connection with Historical Studies in the Arts of Healing and Hygiene

**Published:** 1891-09

**Authors:** 


*Medical Symbolism, in connection with Historical Studies in the Arts of Healing and Hygiene.* By THOMAS S. SOZINSKEY, M.D., Ph.D. Philadelphia: F. A. DAVIS. 1891.

It is unfortunately to Dr. Sozinskey's memory only that we can return thanks for this book. The erudite author himself has been dead for over two years; and his death followed too soon upon the completion of the work for him to witness its publication. In our struggle to be acquainted with the staining properties of the last discovered microbe, we have had no time of late to devote to the study of the history of Medicine, so that it is a novelty as well as a pleasure to peruse again a treatise of this character. Dr. Sozinskey has brought together by much laborious research a large amount of information bearing upon the history of Medicine and Symbolism. His style is pleasant, his language well chosen, and the subject interesting; so that although the book is learned it is easy reading. So far as we know, this is the first medical work in which the "Aryan" origin of learning and belief is not universally accepted, and in which there is also demonstrated how much of existing knowledge and faith we have inherited from the pre-Semitic dwellers in Mesopotamia. To say that Dr. Sozinskey is not at times a little diffuse would not be strictly true, but we most heartily commend the work, while deploring the premature death of the accomplished author.

